# High Insulin Requirements and Poor Metabolic Control do not Modify the Expression, Regulation and PKC Mediated Activation of the p21ras Pathway in PBMC from Type II Diabetic Patients

**DOI:** 10.1155/EDR.2001.47

**Published:** 2001

**Authors:** M. J. Rapoport, O. Levi, M. Weiss, A. Buchs, Y. Ramot, D. Aharoni, A. Mor, G. Elberg, Y. Katz, J. Weissgarten

**Affiliations:** 1 Diabetes and Endocrinology Unit Assaf Harofeh Medical Center the Sackler Facilty of Medicine Tel-Aviv University Zerifin 70300 Israel; 2 Department of Internal Medicine “C” Assaf Harofeh Medical Center the Sackler Facilty of Medicine Tel-Aviv University Zerifin 70300 Israel; 3 Department of Cell Biology Baylor College of Medicine Houston Texas USA; 4 Department of lmmunology Assaf Harofeh Medical Center the Sackler Facilty of Medicine Tel-Aviv University Zerifin 70300 Israel; 5 Department of Nephrology Assaf Harofeh Medical Center the Sackler Facilty of Medicine Tel-Aviv University Zerifin 70300 Israel

## Abstract

*Aims* To asses whether clinically severe insulin resistance and poor metabolic control in patients with type II diabetes are associated with aberrant expression or function of the p21ras pathway.

*Methods* We examined the expression and function of the p21ras pathway in resting and activated PBMC from 10 insulin treated patients with type II diabetes characterized by high insulin requirements and poor metabolic control (IR group) and 10 age and sex matched well controlled patients treated by diet alone or oral hypoglycemic medications (WC group).

*Results* Levels of p21ras and its regulatory elements: p21rasGAP and hSOS1, were comparable in the two groups. The induced activities of p21ras and its associated down-stream regulatory enzyme MAP-kinase following TPA stimulation were also comparable in the IR and WC patients.

*Conclusions* Taken together, these data indicate that clinically significant severe insulin resistance does not modify the expression, regulation and activation of p21ras pathway in PBMC of patients with type II diabetes.


*Int. Jnl. Experimental Diab. Res.*, Vol. 2, pp. 47–54
Reprints available directly from the publisher
Photocopying permitted by license only

© 2001 OPA (Overseas Publishers Association) N.V.
Published by license under
the Harwood Academic Publishers imprint,
part of Gordon and Breach Publishing,
a member of the Taylor & Francis Group.
Printed in the United States.

### High Insulin Requirements and Poor Metabolic Control do not Modify the Expression, Regulation and PKC Mediated Activation of the p21ras Pathway in PBMC from Type II Diabetic Patients

M. J. RAPOPORT[a,b,*], O. LEVI[b], M. WEISS[a], A. BUCHS[a,b], Y. RAMOT[a], D. AHARONI[a], A. MOR[a], G. ELBERG[c], Y. KATZ[d] and J. WEISSGARTEN[e]

[a]*Diabetes and Endocrinology Unit*, [b]*Department of Internal Medicine "C"*, [d]*Immunology*, [e]*Nephrology, Assaf Harofeh Medical Center, Affiliated to the Sackler Faculty of Medicine, Tel-Aviv University, Israel;* [c]*Department of Cell Biology, Baylor College of Medicine Houston Texas USA*

*(Received 22 August 2000; In final form 15 December 2000)*

***Aims*** **To asses whether clinically severe insulin resistance and poor metabolic control in patients with type II diabetes are associated with aberrant expression or function of the p21ras pathway.**
***Methods*** **We examined the expression and function of the p21ras pathway in resting and activated PBMC from 10 insulin treated patients with type II diabetes characterized by high insulin requirements and poor metabolic control (IR group) and 10 age and sex matched well controlled patients treated by diet alone or oral hypoglycemic medications (WC group).**
***Results*** **Levels of p21ras and its regulatory elements: p21rasGAP and hSOS1, were comparable in the two groups. The induced activities of p21ras and its associated down-stream regulatory enzyme MAP-kinase following TPA stimulation were also comparable in the IR and WC patients.**
***Conclusions*** **Taken together, these data indicate that clinically significant severe insulin resistance does not modify the expression, regulation and activation of p21ras pathway in PBMC of patients with type II diabetes.**

*Keywords*: Type II diabetes; Insulin resistance; PBMC; p21ras; ras-GAP; hSOS; PBMC

*Abbreviations*: ECL, Enhanced Chemiluminescence; GAP, GTPase Activating Protein; GlyHb, Glycated Hemoglobin; GNRF, Guanine Nucleotide Releasing Factor; hSOS1, human Son of Sevenless; MAP-kinase, Mitogen Activated Protein kinase; PBMC, Peripheral Blood Mononuclear Cells; PHA, Phytohaematoagglutinin; PKC, Protein Kinase C; TCR, T cell Receptor; TK, Tyrosine Kinase; TPA, 12-0-tetradecanoylphorbol-13-acetate

### INTRODUCTION

The p21ras protooncogenes are a heterologous family of GTP/GDP-binding, growth promoting proteins located downstream of receptor associated TK's in many cell types. The activity of these proteins is regulated by their bound GTP/GDP ratio and is directly proportional to this ratio; GTP-p21ras and GDP-p21ras are the active and the non-active states, respectively. The relative amounts of GTP and GDP bound to p21ras are regulated by two opposing factors.

*Address for correspondence: Assaf Harofeh Medical Center, Zerifin 70300, Israel. Tel.: +972-8-9779282, Fax: +972-8-9779285, e-mail: mrapoport@asaf.health.gov.il

GNRF's, such as SOS, enhance GTP binding to p21ras by accelerating the dissociation of prebound GDP. The 120 kDa p21rasGAP and other GAP-like proteins enhance the relatively weak intrinsic GTPase activity of non-transforming p21ras proteins.[1,2] In lymphoid cells, stimulation of membranal receptors such as the TCR or the IL-2 receptors activates the TK's or the PKC dependent intracellular signaling pathways which mediate p21ras-GAP inhibition and increased hSOS activity resulting ultimately in p21ras activation.[3]

Insulin resistance plays a major role in the pathogenesis and progression of Type II Diabetes. Its cause is unknown in the vast majority of patients and is commonly attributed to post insulin receptor signaling defect.[4] Insulin receptor signaling is mediated in part *via* the p21ras pathway.[5–7] Over-expression of a p21ras like protein called "ras associated with diabetes" (RAD) has been reported in muscle tissue of patients with type II diabetes and implicated as a cause of insulin resistance in these patients.[8] However, genetic studies failed to confirm a linkage between the rad locus and familial predisposition to type II diabetes.[9] Furthermore, we previously reported that the induced activity, as well as expression of p21ras and its regulatory factors were normal in PBMC of insulin treated type II patients and comparable to non-diabetic control patients.[10] In this work, we examined for the first time the function and expression of the p21ras pathway in a subset of these patients characterized by high insulin requirements and poor metabolic control to further determine the role of p21ras pathway in mediating the insulin resistance in type II diabetes.

### PATIENTS AND METHODS

Ten insulin treated type II diabetes patients with severe insulin resistance and poor metabolic control, mean age 58.8 $\pm$ 11.3 years, range 46–68, and 10 WC patients treated with diet alone or oral hypoglycemic medications, mean age 54.8$\pm$6.7 years, range 42–70, participated in the study. Mean disease duration was 15$\pm$4 years and 8$\pm$6 years respectively. Clinical and demographic parameters of these patients appear in Table I. Classification of the patients was made according to the National Diabetes Data Group guidelines.[11] None of the diabetic patients or controls suffered at the time of his enrollment from any other significant acute or chronic disease. Insulin resistance was defined as daily insulin requirements >1 Unit/kg/day. None of the patients in the IR group suffered from any endocrinopathies, metabolic derangements or genetic syndromes associated with clinical insulin resistance. Only one patient from the insulin resistance group had detectable but insignificant low levels of anti-insulin antibodies. All participants signed an informed consent and the hospital ethics committee approved the study. Twenty ml of blood was drawn from an antecubital vein into heparinized tubes from each patient. PBMC were isolated by Ficol-Hypaque gradient centrifugation. Cells were either used immediately for the p21ras/MAPK activity and proliferation assays, or frozen and kept at −70°C for the western blots.

### Antibodies

Rabbit anti-GAP polyclonal antibody was purchased from Santa Cruz Biotechnology (CA, USA). Purified ascites containing Y13-259 anti p21v-H-ras mAb was kindly donated by Dr. G. B. Mills (Toronto General Hospital, Toronto, ON, Canada). The rabbit anti hSOS1 polyclonal Ab was purchased from UBI (Lake Placid NY, USA).

### *In Vitro* Proliferation Assay

$2\times10^5$ cells/well were cultured in RPMI medium supplemented with 5% heat inactivated bovine calf serum, glutamine 2mM, penicillin 100 U/ml, 200mg/dl glucose and streptomycin 100µg/ml (Biological Industries, Beit Haemek, Israel) in

TABLE I Clinical and demographic parameters of the study patients and controls

| Group | Number of patients (m/f) | AGE yrs (mean±SD) | #Disease duration yrs (mean±SD) | Insulin U/Kg/D (mean±SD) | BMI $kg/m^2$ (mean±SD) | ^Glycated **Hb% (mean±SD) |
|---|---|---|---|---|---|---|
| IR | 10 (6/4) | 58.8 ± 11.3 | 15 ± 3.9 | 1.43 ± 0.5 | 30.8 ± 4.9 | 12.3 ± 1.4 |
| WC | 10 (6/4) | 54.8 ± 6.7 | 8 ± 5.9 | – | 30.6 ± 6.9 | 8.2 ± 0.6 |

#P < 0.008.

^P < 0.002.

**Normal range 6.8–8.8%.

0.2ml round-bottomed microtiter plates. Cells were cultured for 3 days in 5% $CO_2$ in air humidified 37°C incubator in the presence of different concentrations of PHA (2.5–20μg/ml). Proliferative response to was assessed by 3H-thymidine uptake (20h pulse) and calculated as stimulation index (maximal proliferation in cpm divided by the background non-stimulated value of each patient).

### Determination of p21ras Activity

Cells ($1 \times 10^7$mL) were permeabilized by addition of 0.4U/mL of streptolysin O (Gibco) and labeled with 5μCi of α-[32P]GTP(5mCi; 3000 Ci/mmol, Amersham), as described.[10] The CHELATE program[12] was used to predict the concentration of $CaCl_2$ and $MgCl_2$ required to give 100nM and 5mm free $Ca^{2+}$ and free $Mg^{2+}$, respectively, at pH 7.2 and 37°C. After stimulation with TPA (10nM) (Sigma Immunochemicals) for 20 min, cells were lysed in ice-cold 50mM Hepes buffer, pH 7.4, containing 1% NP-40, 1mM EGTA, 150mM NaCl, 5mM $MgCl_2$, 1mM PMSF, 10mg/ml leupeptin, 10mg/mL aprotinin, 10mg/ml soybean trypsin inhibitor, 0.5% deoxycholate and 0.05% SDS. Lysates were precleared for 5 min at 4°C with goat anti-rat IgG coupled to agarose (Sigma Immunochemicals). Immunoprecipitations (45min at 4°C) were performed in duplicate using 5μg/ml of either Y13-259 anti p21v-H-ras mAb or normal rat IgG (Jackson Immunoresearch Laboratories, West Grove, PA), followed by goat anti-rat IgG coupled to agarose. After washing (1ml×8), nucleotides were eluted by incubation for 20min at 68°C, and separated on polyethylene-imine-cellulose, thin layer, chromatography plates developed in 1M $KH_2PO_4$, pH 3.4. Plates were autoradiographed and determination of ras-bound GTP/GDP was evaluated by photodensitometry (Computing densitometer, molecular dynamics, Model 300A; Eugene OR).

### Western Blots

$2 \times 10^7$ Cells were lysed in lysis buffer and boiled for 2min in SDS sample buffer. Proteins (50μg/lane) were separated by SDS-PAGE, transferred to nitrocellulose membrane and immunoblotted with either Y13-259 anti-p21v H-ras mAb (4μg/ml), rabbit anti-GAP polyclonal antibody (1μg/ml) or the rabbit anti-hSOS1 polyclonal Ab (4μg/ml). Normal rat IgG or normal rabbit preimmune serum were used as control for the determination of p21ras, 120rasGAP and hSOS1, respectively. Anti-rat and anti-rabbit HRP mAb's were used successively for blotting of p21ras and 120rasGAP, respectively. Membranes were developed with ECL and autoradiographed. Protein expression was determined by photodensitometery (Computing densitometer, Molecular dynamics, Model 300A; Eugene OR).

### MAP-kinase Activity Assay

Determination of MAP-kinase activity was performed as previously described.[13] Briefly, following stimulation cells ($10^7$/treatment) were frozen at −70°C, thawed and homogenized in 50 μl of 50 mM β-glycerol phosphate buffer (pH 7.3) containing 1.5 mM EGTA, 1 mM EDTA, 1 mM dithiotreitol, 0.1 mM sodium vanadate, 10 μg/ml leupeptin, 10 μg/mL aprotinin and 10 μg/ml pepstatin A. The homogenates were centrifuged at 15,000g for 20 min and the supernatants (cytosolic extracts) were supplemented with five fold concentrated sample buffer (300 mm Tris-HCL, pH 6.8, 10% [w/v] SDS, 25% [v/v] glycerol and 0.025% [w/v] pyronine y). A gel shift assay was performed by applying the cytosolic extract on SDS-PAGE using polyacrylamide (10%w/v) and bisacrylamide (0.1%w/v) to obtain optimal separation of the phosphorylated and non-phosphorylated forms of MAP-kinase. The proteins were transferred onto nitrocellulose membrane and reacted with rabbit polyclonal antibodies to MAP-kinase (1:2000 dilution). The enzyme was detected with ECL kit according to manufacturer's instructions.

### Statistical Analysis

Statistical analysis was carried out by Student's t-test for comparisons of means. Differences between groups were considered statistically significant at $p<0.05$.

### RESULTS

### Normal p21ras Expression in IR Patients

To examine the p21ras pathway, we initially determined the levels of p21ras in our insulin resistant and well-controlled patients. Expression of p21ras was comparable in the two groups: 2904±830 and 3012±256 in insulin resistant and well controlled patients, respectively (Fig. 1). No correlation was found between p21ras expression and any of the clinical or demographic parameters of these patients including metabolic control, disease duration or daily insulin requirements (data not shown). These data indicate that severe insulin resistance does not modify p21ras expression in PBMC of patients with type II diabetes.

### Expression of p21ras Regulatory Elements is Normal in Insulin Resistant Patients

To further examine the regulation of p21ras pathway in insulin resistant patients we determined the levels of the p21ras inhibitory element p120rasGAP and the stimulatory factor hSOS1 in these patients. Expression of p21ras-GAP and hSOS1 were comparable in the two groups: 2289±979 and 1833±300 and 625±183 and 731±226 for insulin resistant and well-controlled

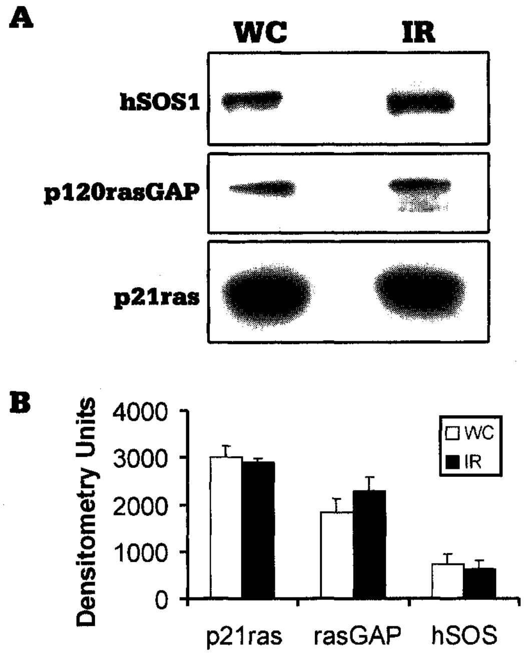

FIGURE 1 Expression of p21ras, p120rasGAP and hSOS1 proteins is comparable in well-controlled (WC) and insulin resistant (IR) type II diabetic patients.

(A) Representative western blots of p21ras, p120rasGAP and hSOS1 from PBMC of well-controlled (WC) and insulin resistant (IR) diabetic patients.

(B) Expression of p21ras, p120 rasGAP and hSOS1 in PBMC lysates of WC (empty bars) and IR (black bars). Protein concentrations were determined by photodensitometry as indicated. Values represent ± SE for each group.

patients, respectively (Fig. 1). These data indicate that the regulation of p21ras pathway in PBMC of type II patients with severe insulin resistant is normally maintained.

### P21ras Pathway Activation in Insulin Resistant Patients

Activation of p21ras pathway may be decreased in the presence of normal expression of p21ras and its regulatory elements.[10,14,15] Therefore, we determined the stimulated p21ras guanine nucleotide binding in the three most insulin resistant patients (mean daily insulin requirements 1.6U/Kg/D±0.5, mean GlyHb 13.1%±1.2) as compared to WC patients (mean GlyHb 7.9%±0.4). Figure 2 shows that TPA stimulated increase in p21ras activity was comparable in insulin resistant and well-controlled patients: 6.45±3.5% and 5.7±7%, respectively. To further analyze this pathway we determined the stimulated activity of p21ras down-stream associated regulatory enzyme MAP-kinase. Exposure to TPA resulted in a similar electrophoretic shift in the phosphorylated 44 kDA form of MAP-kinase: 30.9% ± 5.7 and 28.7% ± 3.5 of total MAP-kinase for IR and WC patients, respectively (Fig. 3). No correlation was found between the degree of p21ras activation and various clinical parameters of these patients including: age, disease duration, insulin requirements in Units/kg/day and

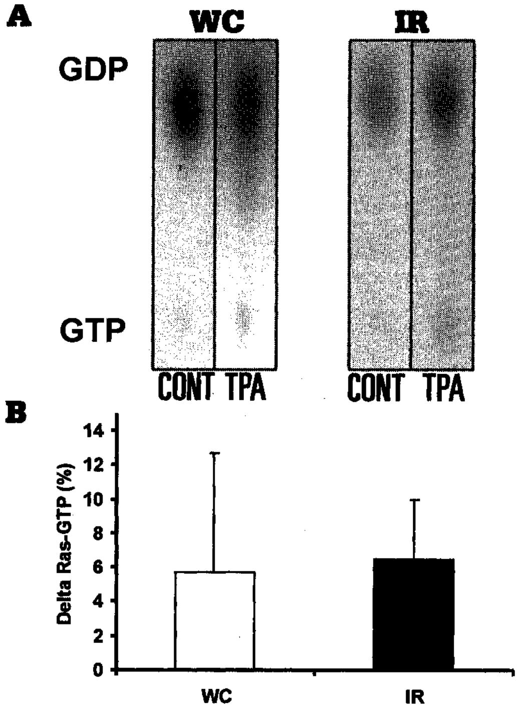

FIGURE 2 p21ras activation in well-controlled (WC) and insulin resistant (IR) type II diabetic patients.

(A) Representative thin layer chromatogram of p21ras-bound nucleotides eluted from PBMC of well-controlled and insulin-resistant patients. Cells were either non-stimulated (CONT) or stimulated with TPA 10ng/ml for 10 min (TPA).

(B) Activation of p21ras in PBMC of WC (empty bars) and IR (black bars) patients. p21ras activation (ΔGTP) was calculated as the percent difference between the ratio of GTP/GDP+GTP of stimulated and non-stimulated cells. Each bar represents mean ± SE of each group.

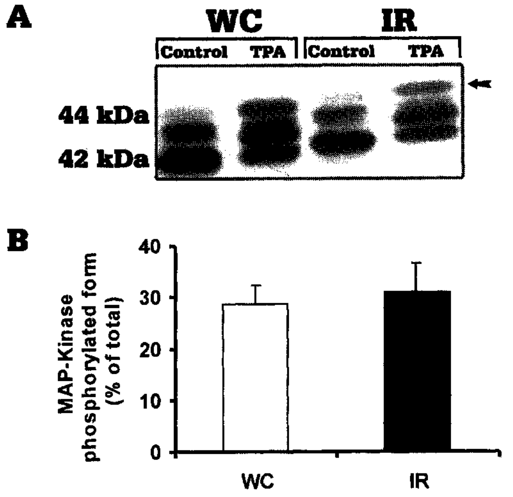

FIGURE 3 Activation of MAP kinase by TPA in PBMC of type II diabetic patients.

Cells from well-controlled (WC) and insulin resistant (IR) patients were exposed to 10nM of TPA for 10min before MAP kinase analysis was performed as described in methods.

(A) Representative immunoblot of cytosolic extract developed with anti MAP kinase antibody. The arrow indicates the electrophoretic shift of MAP kinase induced by TPA.

(B) Densitometric analysis of MAP kinase activation in WC (empty bars) and IR (black bars) patients. Data (mean ± SE of each group) shows the phosphorylated form of MAP kinase expressed as percent of total MAP kinase.

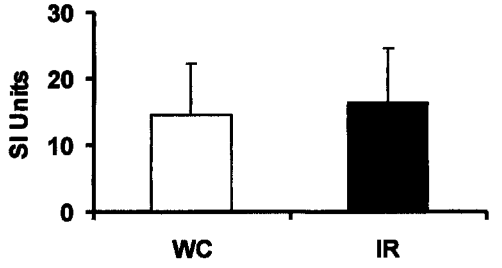

FIGURE 4 Proliferative response of PBMC from well-controlled (WC) and insulin resistant (IR) patients.

PBMC were isolated from WC (empty bars) and IR (black bars) patients as described. Maximal proliferative response to PHA (2.5–20μg/ml) was assessed after three days by $^3$H-thymidine uptake (20 h pulse). Values represent mean ± SE of each group and are expressed in SI units calculated as the maximal proliferation in cpm divided by the background non-stimulated value of each patient.

GlyHb levels. Taken together, these data indicate that the basal and stimulated activities of p21ras pathway is normal in PBMC of type II patients characterized by severe insulin resistance.

### Normal Proliferative Response of PBMC from Insulin Resistant Patients

We previously reported a normal p21ras pathway expression in the presence of defective lymphocyte activation and proliferation.[14] Therefore, we determined the mitogen mediated *in vitro* proliferation of our insulin resistant and well-controlled patients. The mean proliferative response to PHA was comparable in insulin resistant and well-controlled patients: SI of 16.4±8.6 and 14.5±7.8, respectively (Fig. 4). Exposure to insulin alone (1–10nm) did not result in a significant proliferative response in both groups (data not shown).

Thus, severe chronic hyperglycemia and high insulin requirements do not decrease the lymphocyte proliferative response of type II diabetic patients.

### DISCUSSION

Our data demonstrate that the expression of p21ras and its regulatory factors in lymphocytes of poorly controlled type II diabetic patients with high insulin requirements are comparable to well controlled patients. No correlation was found between the expression of the p21ras pathway constituents in these patients and any of their clinical parameters including disease duration, glycated Hb levels and BMI or daily insulin requirements. This suggests that the p21ras pathway is not affected by the long standing severe insulin resistance and the resulting poor metabolic control of patients with type II diabetes. Furthermore, it does not support a role for this pathway in mediating the insulin resistance of these patients. These findings differ from the findings of Reynet et al who have reported increased expression of p21ras like protein namely rad in muscle tissue of type II diabetic patients.[8] This discrepancy could result from the methodological differences between the two studies *i.e.*, protein levels *vs.* mRNA determination or the minor but significant sequence differences between p21ras and rad. It could also result from the different tissues examined *i.e.*, muscle *Vs.* lymphocytes. If this is the case it may indicate that p21ras or p21ras like protein over expression in type II diabetic patients is not an inherent generalized finding in insulin responsive tissues of these patients but is rather tissue specific.

We previously demonstrated that reduced activation of the p21ras pathway is detectable in the presence of normal expression of p21ras and its regulatory factors.[10,14,15] In addition, a variety of signaling defects has been reported in PBMC from type II patients[16–18] and hyperglycemia has been implicated as a reversible cause of aberrant T cell activation in diabetic patients.[19] This raised the question whether p21ras signaling rather than expression is affected by long standing hyperglycemia. Our data demonstrate that TPA mediated activation of p21ras and activation of its downstream regulatory enzyme MAP kinase is intact in the subset of patients with the most severe insulin resistance and comparable to well-controlled patients. This suggests that the functional integrity of the p21ras/MAP kinase pathway is normally maintained in insulin resistant type II patients. This notion is in agreement with the recent demonstration of a normal expression and insulin mediated MAP kinase phosphorylation in isolated skeletal muscle from moderately controlled type II diabetic patients.[20] However, it should be pointed out that we examined only the downstream part of the p21ras pathway. Thus, it remains possible that severe insulin resistance and poor metabolic control affect the early membrane receptor mediated activation of p21ras pathway rather than disrupt its late signaling events. This intriguing possibility is consistent with the reported down-regulating effect of glucose toxicity on early insulin receptor signaling cascade[21,22] and by the recent demonstration that ras transformed brown adipocytes developed insulin resistance due to a signaling defect up-stream of ras, without decreasing the activity of p21ras down-stream elements.[23] It is also supported by our previous demonstration in type I patients and pre-diabetic animals of a restricted defect in the activation of p21ras located up-stream of PKC – p21ras complex and the cell membrane.[10,14,15] It should also be pointed out that we did not provide here data regarding the effect of insulin itself on p21ra/MAP kinase pathway activity in our patients inasmuch as exposure to insulin in physiological concentrations *in vitro* did not result in any detectable increase in their GTP-p21ras (data not-shown). This is consistent with the reported effects of other hormones such as hGH which act *via* the p21ras pathway. The small but detectable effect on the p21ras pathway of the latter is achieved only when its concentration is at least one order of magnitude higher than that necessary for mitogenesis.[13] Furthermore, others have reported that an overexpression of the insulin receptor is necessary to examine the effect of insulin on p21ras pathway activity *in vitro*.[7] Thus, our current experiments do not allow us to decide whether insulin-mediated signaling *via* the p21ras/MAP kinase pathway remains unaltered in our insulin resistant patients.

In conclusion, our data provide evidence for a normal expression and activation of p21ras pathway in PBMC of poorly controlled type II diabetic patients with clinically significant insulin resistance. Further studies with a larger number of patients and examination of a wide range of insulin responsive tissues are necessary to determine the role of the p21ras pathway in mediating insulin resistance in these patients.

### *Acknowledgements*

We thank Ran Yariv, Anji Agajany and Jane Geva for their expert secretarial and technical assistance.

### *References*

[1] Pawson, T. (1995). Protein modules and signaling networks. *Nature*, **373**, 573–580.
[2] Grand, R. J. and Owen, D. (1991). The biochemistry of ras p21. *Biochem. J.*, **279**, 609–631.
[3] Izquierdo, P. M., Reif, K. and Cantrell, D. (1995). The regulation and function of p21ras during T-cell activation and growth. *Immunol Today*, **16**, 159–164.
[4] Kahn, C. R. (1994). Insulin action, diabetogenes, and the cause of Type II diabetes. *Diabetes*, **43**, 1066–1084.
[5] Korn, L. J., Sieble, C. W., McCormick, F. and Roth R. A. (1987). Ras p21 as a potential mediator of insulin action in *Xenopus* oocytes. *Science*, **236**, 840–843.
[6] Kozma, L., Baltensperger, K., Klarlund, J., Porras, A., Santos, E. and Czech, M. P. (1993). The *ras* signaling pathway mimics insulin action on glucose transporter translocation. *Proc. Natl. Acad. Sci. USA*, **90**, 4460–4464.
[7] Burgering, B. M., Medema, R. H., Maassen, J. A., van de Wetering, M. L., van der Eb, A. J., McCormick, F. and Bos, J. L. (1991). Insulin stimulation of gene expression mediated by p21ras activation. *EMBO J.*, **10**, 1103–1109.
[8] Reynet, C. and Kahn, C. R. (1993). RAD: a member of the Ras family over-expressed in muscle of type II diabetic humans. *Science*, **262**, 1441–1444.
[9] Elbein, S. C., Chiu, K. C., Hoffman, M. D., Mayorga, R. A., Bragg, K. L. and Leppert, M. F. (1995). Linkage Analysis of 19 Candidate Regions for Insulin Resistance in Familial NIDDM. *Diabetes*, **44**, 1259–1265.
[10] Rapoport, M. J., Mor, A., Vardi, P., Ramot, Y., Levi, O. and Bistritzer, T. (1998). Defective activation of p21ras in peripheral blood mononuclear cells from patients with Insulin Dependent Diabetes Mellitus. *Autoimmunity*, **29**, 147–154.

[11] National Diabetes Data Group. (1979). Classification and diagnosis of diabetes mellitus and other categories of glucose intolerance. *Diabetes*, **28**, 1039–1057.

[12] Graves, J. D., Lucas, S. C., Alexander, D. R. and Cantrell, D. A. (1990). Guanine nucleotide regulation of inositol phospholipid hydrolysis and CD3-antigen phosphorylation in permeabilized T lymphocytes. *Biochem. J.*, **265**, 407–413.

[13] Elberg, G., Rapoport, M. J., Vashdi-Elberg, D., Gertler, A. and Shechter, Y. (1996). Lactogenic hormones rapidly activate p21ras/mitogen-activated protein kinase in Nb2-11C rat lymphoma cells. *Endocrine*, **4**, 65–71.

[14] Rapoport, M. J., Lazarus, A. H., Jaramillo, A., Speck, E. and Delovitch, T. L. (1993). Thymic T cell anergy in autoimmune non obese diabetic mice is mediated by deficient T cell receptor regulation of the pathway of p21ras activation. *J. Exp. Med.*, **177**, 1221–1226.

[15] Rapoport, M. J., Weiss, L., Mor, A., Bistritzer, T., Ramot, Y. and Slavin, S. (1996). Prevention of autoimmune diabetes by Linomide in NOD mice is associated with upregulation of the T cell receptor mediated activation of p21ras. *J. Immunol.*, **157**, 4721–4725.

[16] Mandarino, L. J., Campbell, P. J., Gottesman, I. S. and Gerich, J. E. (1984). Abnormal coupling of insulin receptor binding in non-insulin dependent diabetes. *Am-J. Physiol.*, **247**, 688–692.

[17] Frittitta, L., Grasso, G., Munguira, M. E., Vigneri, R. and Trischitta, V. (1993). Insulin receptor tyrosine kinase activity is reduced in monocytes from non-obese normoglycaemic insulin-resistant subjects. *Diabetologia*, **36**, 1163–1167.

[18] Curto, M., Novi, R. F., Rabonne, I., Maurino, M., Piccinini, M., Mioletti, S., Mostert, M., Bruno, R. and Rinaudo, M. T. (1997). Insulin resistance in obese subjects and newly diagnosed NIDDM patients and derangement's of pyruvate dehydrogenase in their circulating lymphocytes. *Int. J. Obes. Relat. Metab. Disord.*, **21**, 1137–1142.

[19] Selam, J. L., Clot, J., Andary, M. and Mirouze, J. (1979). Circulating lymphocyte subpopulations in juvenile insulin-dependent diabetes. Correction of abnormalities by adequate blood glucose control. *Diabetologia*, **16**, 35–40.

[20] Krook, A., Bjornholm, B., Galuska, D., Jiang, X. J., Fahlman, R., Myers Jr., M. G., Wallberg-Henriksson, H. and Zierath, J. R. (2000). Characterization of signal transduction and glucose transport in skeletal muscle from type 2 diabetic patients. *Diabetes*, **49**, 284–292.

[21] Ide, R., Maegawa, H., Kashiwagi, A., Kikkawa, R. and Shigeta, Y. (1995). High glucose condition desensitizes insulin action at the levels of receptor kinase. *Endocr. J.*, **42**, 1–8.

[22] Kroder, G., Bossenmaier, B., Kellerer, M., Capp, E., Stoyanov, B., Muhlhofer, A., Berti, L., Horikoshi, H., Ullrich, A. and Haring, H. (1996). Tumor necrosis factor – alpha – and hyperglycemia – induced insulin resistance. Evidence for different mechanisms and different effects on insulin signaling. *J. Clin. Invest.*, **97**, 1471–1777.

[23] Valverde, A. M., Lorenzo, M., Teruel, T. and Benito, M. (1997). Alterations in the insulin signaling pathway induced by immortalization and H-ras transformation of brown adipocytes. *Endocrinology*, **138**, 3195–3206.

